# In Vitro and In Vivo Models for the Study of Human Polyomavirus Infection

**DOI:** 10.3390/v8100292

**Published:** 2016-10-22

**Authors:** Heidi Barth, Morgane Solis, Wallys Kack-Kack, Eric Soulier, Aurélie Velay, Samira Fafi-Kremer

**Affiliations:** 1 Laboratoire de Virologie, Hôpitaux Universitaires de Strasbourg, 3 rue Koeberlé, 67000 Strasbourg, France; morgane.solis@chru-strasbourg.fr (M.S.); wallys.kack-kack@chru-strasbourg.fr (W.K.-K.); aurelie.velay@chru-strasbourg.fr (A.V.); samira.fafi-kremer@chru-strasbourg.fr (S.F.-K.); 2 Université de Strasbourg, INSERM, IRM UMR-S 1109, 4 rue Kirschleger, 67000 Strasbourg, France; eric.soulier@unistra.fr

**Keywords:** polyomavirus, in vitro models, animal models, tropism, entry, pathogenesis

## Abstract

Developments of genome amplification techniques have rapidly expanded the family of human polyomaviruses (PyV). Following infection early in life, PyV persist in their hosts and are generally of no clinical consequence. High-level replication of PyV can occur in patients under immunosuppressive or immunomodulatory therapy and causes severe clinical entities, such as progressive multifocal leukoencephalopathy, polyomavirus-associated nephropathy or Merkel cell carcinoma. The characterization of known and newly-discovered human PyV, their relationship to human health, and the mechanisms underlying pathogenesis remain to be elucidated. Here, we summarize the most widely-used in vitro and in vivo models to study the PyV-host interaction, pathogenesis and anti-viral drug screening. We discuss the strengths and limitations of the different models and the lessons learned.

## 1. The Growing Family of Human PyV

Mouse polyomavirus (PyV), discovered in the 1950s, was the founder of the PyV family. It was termed polyoma (meaning “many tumors”)-virus because of its ability to produce tumors in mice [1]. In 1971, the first two human PyV were discovered independently in specimens from immunocompromised patients and were named after the patients’ initials: BK and JC. BK polyomavirus (BKPyV) was isolated from the urine of a kidney transplant recipient [2], and JC polyomavirus (JCPyV) was identified in the brain tissue of a patient with a history of Hodgkin’s lymphoma and progressive multifocal leukoencephalopathy [3]. However, over 35 years passed before advances in high-throughput sequencing technologies were to suddenly increase the discovery of new human PyV (Table 1). In 2007, Karolinska Institute (KI) PyV [4] and Washington University (WU) PyV [5] were discovered by means of the random polymerase chain reaction (PCR) amplification and high-throughput deoxyribonucleic acid (DNA) sequencing strategy involving respiratory samples from patients with acute respiratory tract infections. In 2008, Merkel cell PyV (MCPyV) was discovered by analyzing complementary DNA (cDNA) sequences prepared from Merkel cell carcinoma specimens using pyrosequencing technology followed by the subtraction of human reads so as to identify novel viral sequences [6].

Rolling circle amplification (RCA) techniques identified human polyomavirus 6 (HPyV6) and HPyV7 from skin samples [7], and trichodysplasia spinulosa-associated PyV (TSPyV, also called HPyV8) in skin lesions from patient with trichodysplasia spinulosa [8]. In 2011, a generic PyV PCR assay using primers that have been designed to target conserved regions of the gene encoding the major capsid protein of PyV led to the discovery of HPyV9 in a kidney transplant patient [9]. Only one year later, Malawi PyV (MWPyV) was identified from stool specimens of a one-year-old healthy Malawi child using RCA and pyrosequencing [10]. In the same year, the complete sequenced genome of HPyV10 was detected in condyloma specimens from a patient with a rare genetic disorder known as warts, hypogammaglobulinemia, infections and myelokathexis syndrome (WHIM) [11]. In addition to these cases, the isolation of a PyV from stool samples of a Mexican child presenting with diarrhea has also been described (Mexico PyV; MXPyV) [12]. An analysis of the nucleotide sequences of MWPyV, HPyV10 and MXPyV isolates revealed 95%–99% homology, suggesting that these PyV are closely-related variants. In 2013, Saint Louis PyV (STLPyV) was identified in the stool sample of a healthy child [13], with nucleotide sequence analysis demonstrating that STLPyV is most closely related to MWPyV [13]. Generic PyV PCR targeting the major viral capsid protein also identified a novel PyV in human liver tissue, called HPyV12 [14]. Phylogenetic analyses of HPyV12 did not reveal a close relationship with known human PyV, indicating that HPyV12 belongs to a different PyV species. The last novel PyV was identified in a muscle biopsy of a pancreatic transplant recipient suffering from retinal blindness and vasculitic myopathy, using high-throughput sequencing, which was named New Jersey PyV (NJPyV). This variant appears to exhibit a tropism for vascular endothelial cells [15].

## 2. The PyV Life Cycle

**Figure 1:**
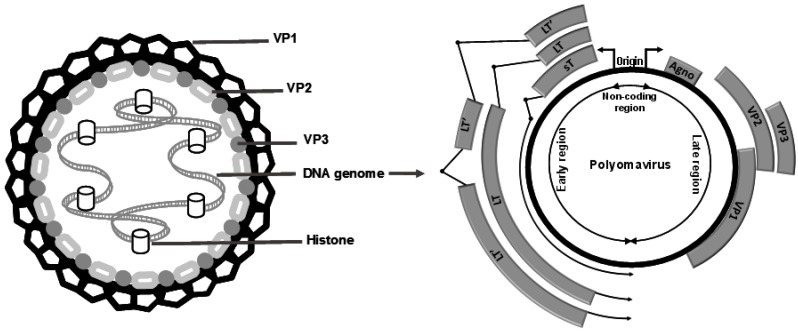
Human PyV particles are composed of 72 pentamers of the capsid protein VP1, with one of the minor capsid proteins VP2 or VP3 in the center of each pentamer. The human PyV genome is divided into three regions: a non-coding region, containing the early and late promoters, transcription sites and the origin of replication; an early region encoding small T antigens (sT), large T antigens (LT) and alternatively-spliced LT antigens (LT’); and a late region encoding the viral structural proteins VP1, VP2 and VP3. Among human polyomaviruses, only BKPyV and JCPyV encode an agnoprotein (agno) upstream of VP1. Merkel cell PyV (MCPyV) does not encode the minor capsid protein VP3.

Human PyV are small non-enveloped viruses. The genome of PyV consists of an ~5 kb-long circular double-stranded DNA and is encapsidated in an icosahedral shell composed of 72 pentamers of the capsid protein VP1. VP1 determines antigenicity and receptor specificity and has thus a significant impact on the attachment, tissue tropism and pathogenicity of PyV. In addition to the major capsid protein VP1, two minor capsid proteins, VP2 and VP3, occupying the interior of the capsid, have been described [16] (Figure 1). Interestingly, MCPyV lacks the VP3 minor capsid protein, and phylogenetic analyses indicate that MCPyV is a member of a divergent clade of polyomaviruses that lack the conserved VP3 N-terminal motif [30]. The genome of all PyV contains an ~500-bp non-coding sequence referred to as the non-coding control region (NCCR), which harbors the origin of DNA replication, as well as transcription promoters and regulatory elements. The NCCR region separates the PyV genome into the early region encoding the tumor antigens (TAgs), which are synthesized before viral DNA replication, and the late region encoding the viral capsid proteins VP1, VP2 and VP3 (Figure 1). TAgs are expressed from a variably-spliced viral transcript resulting in different forms of TAgs (large (LTAg), middle (MTAg) and small (sTAg) forms). Unlike the other human PyV, BKPyV and JCPyV encode an accessory protein in the late region, referred to as the agnoprotein [16]. In addition, viral encoded miRNAs, which have the ability to negatively regulate the expression of viral gene expression, have been found to be encoded by BKPyV, JCPyV and MCPyV [31,32,33,34].

PyV entry is initiated by the major capsid protein VP1, which attaches to cellular receptors to promote internalization. Sialylated glycans have been identified as functional receptors for several human PyV. Sialic acids are abundantly expressed on *N*- or *O*-linked glycoproteins and gangliosides. BKPyV uses the common α2,8-disalic acid motif on b-series gangliosides to infect cells [35,36]. Although JCPyV binds to multiple sialic acid-containing gangliosides, the linear α2,6-linked lacto-series tetrasaccharide c (LSTc) has been established as a functional receptor for JCPyV [37,38]. In addition to LSTc, other studies have also evidenced the importance of other cellular components for JCPyV entry, such as the serotonin receptor 5HT2A [39,40]. Interestingly, TSPyV can interact with terminal sialic acids in α2,3-, α2,6- and α2,8-linkages, and a sialylated glycolipid has been proposed to initiate viral entry [41]. Glycan microarray analysis revealed that HPyV9 VP1 also interacts with sialic acids with an unexpected preferential binding to α-5-*N*-glycolylneuraminic acid (Neu5Ac), which humans can acquire only from diet sources that are rich in Neu5Ac, such as red meat and milk products [42]. Unlike other PyV, MCPyV uses sulfated carbohydrates termed glycosaminoglycans as attachment receptors and sialylated glycans as secondary, post-attachment co-receptors during viral entry [43,44]. To date, the cell surface receptors for KIPyV, WUPyV, HPyV6 and HPyV7 are unknown. Single-cell binding studies indicated that sialylated glycans are likely not required for viral attachment of HPyV6 and HPyV7 [45]. Structural analysis of the major capsid protein VP1 revealed that KIPyV and WUPyV VP1 possess unique structural features that suggest engagement of non-sialylated receptor types [46]. Different entry pathways have been described for PyV depending on both cell type and virus. JCPyV virus enters cells via clathrin-mediated endocytosis, while for BKPyV, caveolae-dependent endocytosis and the caveolin- and clathrin-independent entry pathway have been observed [47,48]. However, further studies are warranted to gain a deeper insight into the cellular receptors and entry pathways of known and newly-discovered PyV. Following entry, PyV have to traffic from the cytoplasm to the nucleus, where the uncoated genome is accessible to the replication machinery of the host cell. TAgs are required for viral replication, cellular transformation or tumorigenicity. Virion assembly occurs in the nucleus; however, very little is known about the egress of viral particles. PyV establish a life-long persistence that is probably achieved by a very low level of viral replication. Interestingly, PyV genomes have been shown to be associated with histone proteins [49,50], suggesting that PyV genomes are susceptible to epigenetic regulation.

## 3. PyV-Associated Pathologies

PyV are ubiquitous, clinically-silent human pathogens indicating that PyV and their hosts establish a symbiotic relationship, although it remains unclear to what extent these two partners benefit. A respiratory route of transmission of PyV has been hypothesized [51]. However, PyV have also been detected in different water environments, including swimming pool waters [52,53], implying that PyV are disseminated through fecal or urine contamination of water. Primary infection is usually asymptomatic and occurs in childhood or during adolescence. A recently-conducted seroepidemiology study of 10 human polyomaviruses in the U.S. population demonstrated that all participants were seropositive for at least one PyV, with a mean of 7.3 PyV and with seroprevalences ranging from 17.6% (for HPyV9) to 99.1% (for HPyV10) [20]. PyV establish a silent, persistent infection in various organs and tissues, such as the urogenital tract, kidneys, the bone marrow, the skin and the brain. Diseases associated with PyV have been so far exclusively described in immunocompromised individuals or patients with immunological abnormalities. Current evidence indicates that PyV-specific T cells and also neutralizing antibodies play a crucial role in the control of PyV replication and recovery from PyV-associated diseases. Specific direct antiviral molecules against PyV are lacking. Thus, patient survival is mainly dependent on the reconstitution of PyV-specific T-cell response. For more detailed reviews on these topics, the reader is referred to other studies [54,55,56,57]. At present, five members of the human PyV family have been associated with specific pathologies (Table 1):

BKPyV is the causative agent of BKPyV-associated nephropathy in kidney transplant recipients and hemorrhagic cystitis in bone marrow transplant patients. Primary sites for BKPyV replication are the renal and uro-epithelium, resulting in lytic destruction of these cells. Replication of BKPyV has been observed under all combinations of immunosuppression [55].

JCPyV causes progressive multifocal leukoencephalopathy (PML), a rapidly-progressive and fatal demyelinating disease. JCPyV replicates in oligodendrocytes and to a lesser extent in astrocytes, leading to demyelinated lesions accompanied by progressive accumulation of neurological deficits and ultimately death. JCPyV causes PML in immunocompromised patients, such as patients with HIV/AIDS, hematological malignancies and in patients receiving immunomodulatory medication, such as integrin very late antigen-4 (VLA-4) monoclonal antibody (natalizumab, Tysabri^®^), leukocyte function associated antigen 1 (LFA-1) monoclonal antibody (efalizumab, Raptiva^®^) and CD20 monoclonal antibody (rituximab, MabThera^®^) for the treatment of multiple sclerosis, Crohn’s disease, lymphoma, severe forms of plaque-type psoriasis and rheumatic diseases [58]. In addition to PML, JCPyV can cause other neurological disorders, such as JCPyV granule cell neuronopathy, JCPyV encephalopathy and meningitis [59].

TSPyV causes the rare skin disease trichodysplasia spinulosa (TS) affecting solid-organ transplant patients undergoing immunosuppressive therapy, especially kidney (and kidney-pancreas) and heart transplant recipients, as well as lymphocytic leukemia patients. The disease is characterized by the development of follicular papules and keratin spines (spicules), predominantly in the face, often accompanied by alopecia of the eyebrows and eyelashes. Histologically, TS is characterized by an abnormal maturation and marked distention of the hair follicles. The inner root sheath cells are highly proliferative and contain excessive amounts of trichohyalin and intraepithelial viral inclusions [60,61].

MCPyV was discovered as the causative agent of Merkel cell carcinoma (MCC), an aggressive neuroendocrine skin cancer with high rates of recurrence, metastatic spread and mortality. Primary risk factors for MCC development include immunosuppression, ultraviolet (UV) light exposure and advanced age [62]. Among the 13 human PyV, MCPyV is the only one that has been clearly associated with cancer in humans. MCPyV DNA is found clonally integrated in the tumor genome of MCC with persistent expression of LTAg and sTAg. LTAg isolated from tumors typically contains a truncated form of LTAg that is functionally incapable of supporting viral replication. Although MCPyV is often present on healthy human skin, efforts to determine the natural host cell type that supports MCPyV infection have only very recently succeeded. Liu and colleagues demonstrated that dermal fibroblasts in human skin support the full MCPyV life cycle [63].

Most recently, HPyV7 was associated with pruritic rash and viremia in lung transplant recipients on immunosuppressive therapy [64]. Rennspiess and colleagues reported the detection of HPyV7 DNA and LTAg expression in human thymic epithelial tumors [65]. Although further studies are necessary to investigate these associations, it is not unlikely that HPyV7, as well as other recently-discovered PyV might be associated with novel pathogenicity in immunocompromised individuals.

## 4. Experimental Model Systems to Study PyV Infection

In vitro and in vivo models are instrumental for the understanding of the viral life cycle, the pathogenicity and the identification of compounds with antiviral activity. Recombinant proteins, virus-like particles, virus pseudoparticles and cell culture-derived infectious viruses are most widely used to recapitulate the viral life cycle, to evaluate the prevalence of virus-specific antibodies and to identify inhibitors that target all aspects of the viral life cycle. In contrast, animal models are important tools for investigating the pathological mechanisms underlying virus-associated diseases and for preclinical testing of antiviral drugs or vaccines.

### 4.1. VP1 Pentamers, PyV-Like Particles and PyV Pseudoparticles

Pentamers of the PyV capsid protein VP1, obtained from bacterial expression vectors encoding for VP1, represent the smallest in vitro model. For example, VP1 pentameric subunits have been used successfully to recapitulate early events in JCPyV and TSPyV infection, including receptor specificity, intracellular trafficking routes of PyV, as well as structural studies of VP1-receptor complexes [37,41,66]. Since the insights gained with VP1 pentamers were in line with the findings obtained with infectious PyV, VP1 pentameric subunits represent an important tool for recently-discovered PyV that are difficult to propagate in cell culture.

PyV-like particles (PyVLP) and PyV pseudoparticles (PsV) consist of stable self-assembled capsid proteins without viral DNA. Yeast and baculovirus expression systems have been used to efficiently produce PyVLP [67]. In these systems, the expression of the major capsid protein VP1 results in self-assembled VP1-derived PyVLP [68,69,70,71]. Microplate enzyme immunoassay coated with PyVLP allowed studies on PyV seroprevalence rates [72,73,74,75]. At present, JCPyVLP are used in a second-generation enzyme-linked immunosorbent assay (ELISA) to detect anti-JCPyV antibodies in human serum [76]. This assay is in clinical use as a risk stratification tool for PML in patients with natalizumab treatment [76,77]. Similarly, a MCPyVLP-based immunoassay has been developed to detect anti-VP1 MCPyV antibodies and to evaluate their prognostic value in patients with MCC [78].

PsV are produced by transfecting human cell lines with expression vectors that encode PyV capsid proteins. Production of BKPsV, JCPsV, MCPsV and TSPsV has been reported using the embryonic kidney-derived cell line 293TT [41,44,79]. Co-transfection with vectors that encode a reporter protein, such as green fluorescent protein (GFP) or Gaussia luciferase, enables rapid quantification of PsV entry into target cells. PsV have turned out to be a valuable tool for the analysis of the early steps of the PyV life cycle, such as viral attachment and entry. Using MCPsV, Schowalter and colleagues [44] demonstrated that MCPyV uses glycosaminoglycans as initial attachment receptors followed by an interaction with sialylated host cell factors. In an effort to gain a deeper insight into the cellular tropism of PyV, 60 human tumor cell lines were transduced with MCPsV and BKPsV, respectively. MCPsV and BKPsV efficiently transduced many of the same cell types, but also many distinct cell types; however, no clear preference for a tissue type emerged [80]. Though PsV are instrumental in deciphering virus interactions with cell surface receptors, they cannot be used to identify the cellular factors required for virus replication and assembly in the host cells. Recently, JCPsV have been used to study the possible inhibitory effects of chemical compounds mimicking cell surfaces molecules involved in JCPyV binding, such as sialic acids or lacto-series tetrasaccharide [81,82]. Interestingly, several molecules with inhibitory activity have been identified and may pave the way for the development of novel anti-JCPyV therapeutic strategies. On the other hand, BKPsV representing different genotypes have been generated to study the activity of neutralizing antibodies from sera of healthy human subjects, kidney transplant recipients and commercially available human immune globulin preparations designed for intravenous immunoglobulin (IVIG) therapy [83,84,85]. Results of these high-throughput serological neutralization studies demonstrated that BKPyV genotypes are distinct serotypes, suggesting that the absence of neutralizing antibodies against a different BKPyV genotype may favor BKPyV replication in the transplant recipient [83,84]. Analysis of neutralizing antibodies present in immunoglobulin preparations showed that they were able to neutralize all BKPyV genotypes [85]. Prospective cohort studies are now warranted to evaluate the potential protective role of BKPyV neutralizing antibodies in BKPyV-associated diseases. Finally, PsV-based neutralization assays have been used to assess the induction of the antibody responses of potential PyV vaccine candidates. Recently, a PML patient has been vaccinated with JCPyVLP, and the induced humoral immune response was assessed by JCPsV-based neutralization assays. After the vaccination, high neutralizing antibody titers against wildtype and mutant JCPyV were observed, and the increase of the neutralizing antibody response was associated with an arrest of PML lesion progression [86].

### 4.2. Cell Culture-Derived Infectious PyV

Attempts to efficiently propagate PyV in cell culture have so far succeeded for BKPyV, JCPyV and MCPyV. For BKPyV and JCPyV, different genetic types have been described, the so-called archetype and rearranged variant. Transient shedding of BKPyV or JCPyV in the urine of healthy individuals demonstrates the presence of the archetype, in which the NCCR is a highly conserved DNA sequence block. It is thought that the archetype is contracted in childhood and then establishes persistent infection in the host. High-level replication of BKPyV and JCPyV has been observed in immunocompromised individuals and is associated with the emergence of the rearranged variant. Rearranged variants of BKPyV and JCPyV are characterized by duplications and deletions of DNA sequence blocks in the NCCR, which enhances viral replication and assembly, leading to enhanced cytopathology. Archetype and rearranged variants of BKPyV and JCPyV can be efficiently propagated in the 293TT human embryonic kidney cell line [87]. Following transfection of episomal PyV DNA into 293TT cells, viral replication, expression of capsid proteins and production of infectious progeny have been demonstrated. Why do 293TT cells support efficient propagation of BKPyV and JCPyV? 293TT cells carry an integrated copy of the simian PyV SV40 genome and a plasmid encoding the cDNA of the simian PyV SV40 LTAg [88]. Constitutive expression of simian PyV SV40 LTAg in these cells drives efficient BKPyV and JCPyV DNA replication from their origins of replication. Interestingly, 293TT cells failed to support replication of other PyV, such as MCPyV, KIPyV and WUPyV. The absence of significant replication of these viruses in 293TT cells can be probably explained by the fact that the simian PyV SV40 LTAg coding sequence has a higher similarity to the LTAg of BKPyV and JCPyV than to the LTAg of MCPyV, KIPyV and WUPyV [87]. Indeed, efficient propagation of MCPyV has been reported in 293TT cells co-transfected with plasmids carrying LTAg and sTAg from MCPyV, named 293-4T cells [44]. The observation that MCPyV-specific TAg expression is a limiting factor in MCPyV propagation may help to develop PyV-permissive cell-culture systems for other, yet unculturable PyV.

Although 293TT cells provide a convenient cell culture system for large-scale propagation of PyV, these transformed cells do not represent a suitable model for the study of the viral life cycle in the natural host cell type. For example, in patients with BKPyV-associated diseases, bladder and renal tubular epithelial cells are the major sites of BKPyV replication. Thus, human renal proximal tubule epithelial cells (RPTEC) are currently used as the state-of-the-art model to study BKPyV infection in vitro [89]. However, RPTEC enter into replicative senescence, which restricts their proliferative potential and the experimental design. Recently, Justice and colleagues [90] assessed host nuclear proteomic changes in BKPyV-infected RPTEC and demonstrated that the host cell DNA damage response signaling and DNA repair pathways were among the most affected host proteins. This study confirmed the results of previous studies with RPTEC indicating that BKPyV usurps proteins implicated in cell-cycle control, DNA replication and repair for efficient viral gene expression and replication [91,92]. This is not surprising because PyV must overcome their limited genome coding capacity and, therefore, rely heavily on the host cellular machinery to replicate their genome. Interestingly, rearranged BKPyV has been shown to replicate in a wide range of different cell types, such as human ovarian, brain and melanoma cancer cell lines, monkey kidney cell lines, i.e., Vero and CV-1 [80,93], human salivary gland cells [93,94] and human fetal lung fibroblast (MRC-5) cells (Barth H. and Soulier E. unpublished observation). A link between BKPyV and the respiratory tract is of particular interest since respiratory transmission routes have been proposed for PyV [51].

In vivo, JCPyV has a major tropism for glial cells and infects productively mainly oligodendrocytes and, to a lesser extent, astrocytes. Various differentiation protocols have been developed to obtain oligodendrocytes and their progenitors from human embryonic stem cells (hESC) [95]. hESC-derived oligodendrocytes are susceptible to cell-culture derived JCPyV [96]. However, the differentiation of oligodendrocytes from hESC remains a challenge, and therefore, the human fetal glial cell line SVG, which constitutively expresses simian PyV SV 40 LTAg, has been mostly used to study JCPyV infection. The SVG cell line was established in 1985 [97] and has been provided by the American Type Culture Collection (ATCC) since 1987. In 2014, Henriksen and colleagues [98] reported that a subpopulation of SVG p12 cells provided by ATCC is productively infected with BKPyV and that BKPyV was present in SVG p12 cells at least since 2006. Since BKPyV and JCPyV are closely related and share up to 70% nucleotide sequence identity, the results obtained in these cells should be taken with caution because the presence of BKPyV may have influenced the results on JCPyV infection. The SVG-A cell line, another subclone of the original SVG human glial cell line, did not contain BKPyV and can be used as an alternative SVG cell line [98]. JCPyV archetype and rearranged variants replicate also efficiently in COS-7 cells (CV-1 cells transformed by an origin-defective mutant of simian PyV SV40 which encodes wildtype T antigen).

Cell culture-derived infectious BKPyV and JCPyV and their physiologically-relevant primary cell culture models are most widely used to study the interplay between PyV and the host cell. Application of these models in routine drug screening processes is limited due to the lack of fluorescent-labelled recombinant PyV. Current antiviral screens with cell culture-derived infectious PyV consist of quantification of PyV genomes in cells by real-time quantitative PCR (qPCR) or the detection of PyV capsid proteins by immunofluorescence staining, which are time consuming and labor intensive. Using the immunofluorescence approach, Brickelmaier and colleagues [99] screened 2000 approved drugs for their anti-JCPyV activity in SVG-A cells and identified 14 potential drug candidates. However, the production of recombinant PyV strains carrying a reporter gene that remains replication-competent and produces viral progeny might facilitate anti-viral drug screening. Recently, Dang and colleagues [100] developed a JCPyV construct containing the iLOV gene, a 336-bp improved light, oxygen or voltage-sensing domain of the plant phototropin gene. Insertion of the iLOV gene into the JCPyV genome allowed full viral replication and production of viral particles in 293FT human embryonic kidney cells. The utility of JCPyV-iLOV has now to be validated in physiologically-relevant primary cell culture models.

### 4.3. Animal Models

The development of animal models to study PyV infection is hampered by the narrow host range and cell specificity of these viruses. For example, inoculation of JCPyV into mice or hamsters resulted in tumor formation, but did not recapitulate the demyelinating disease caused by JCPyV in humans [101]. Currently-available small animal models for PyV infection include transgenic models, xenografts, humanized mice and infectious mouse PyV models.

#### 4.3.1. Transgenic Mouse Model

Several transgenic mice harboring the early region of PyV have been used to study the role of TAgs in the pathogenesis of PyV-associated diseases. Transgenic mice expressing JCPyV TAgs in all cells exhibited a shaking disorder and dysmyelination in the central nervous system, but not the peripheral nervous system [102]. Furthermore, expression of TAgs in oligodendrocytes arrested the maturation of oligodendrocytes and the production of myelin [103,104]. However, other phenotypes of JCPyV TAg transgenic mice have also been reported, such as adrenal neuroblastomas and malignant peripheral nerve sheath tumors [105,106], suggesting that the genetic background of the mice and the choice of the promoter that drives the expression of the transgene influence the disease phenotype. Similarly, transgenic mice containing the early region of BKPyV developed primary hepatocellular carcinomas and renal tumors [105], but did not recapitulate key characteristics of BKPyV-associated nephropathy. Spurgeon and colleagues [107] therefore used an inducible transgenic mouse model and a keratin 14 promoter, which allows for skin-specific expression of truncated LTAg and wild-type sTAg. Expression of MCPyV TAgs in stratified squamous epithelial cells and Merkel cells of the skin epidermis led to the development of benign epithelial tumors, but not MCC. Inducible expression of MCPyV sTAg under the control of a keratin 5 promoter led to squamous cell carcinoma-like lesions [108], indicating that sTAg has the capability for epithelial transformation independent of LTAg. However, lesions resembling MCC either morphologically or biochemically by staining with MCC markers were not detected. Similarly, Shuda and colleagues [109] demonstrated in their transgenic mouse model that sTAg expression induces cell proliferation, but was insufficient to recapitulate MCC. Although these robust transgenic mouse models allow insights into the oncogenic potential of TAgs and their potential cellular interaction partners, they have failed to phenotypically recapitulate the human PyV-associated diseases.

#### 4.3.2. Xenograft Mouse Model

Conventional xenograft mouse models consist of the transplantation of cultured human cells in immunocompromised host mice [110]. Transplanted MCPyV-positive MCC cell lines into immunodeficient mice formed tumors, which stained positive for LTAg and cytokeratin-20 (CK20), a marker commonly used in the diagnosis of MCC [111]. Although human xenograft models have limited applications in the study of the mechanisms of PyV pathogenesis, they can be used as predictive preclinical models for new anticancer agents. For example, YM155, a small-molecule inhibitor of survivin, has been tested in MCPyV-positive MCC xenografts. YM155 was non-toxic and led to growth arrest of MCC tumors in these mice [111,112].

#### 4.3.3. Humanized Mouse Models

Mice bearing human tissues, including functional human immune systems, are valuable tools to study the pathogenesis of human-specific infectious pathogens. Tan and colleagues [113] engrafted immunodeficient mice with human lymphocytes and thymus to study JCPyV infection. However, mice intraperitoneally inoculated with brain-derived rearranged JCPyV isolate Mad4 or the urine-derived archetype strain did not show signs or symptoms of PML. JCPyV DNA was occasionally detected in blood and urine, and only in a subgroup of mice, anti-JCPyV humoral and cellular immune responses were induced at low levels [113]. The lack of a robust JCPyV replication and pathogenesis in these mice is likely due to the absence of human brain tissue, the cellular target for JCPyV replication. Kondo and colleagues [114] therefore generated a mouse model with humanized glia cells by implanting primary human glial progenitor cells into neonatal immunodeficient and myelin-deficient (Rafg2-/- Mbp shi/shi) mice. The transfer of human glial progenitor cells resulted in a differentiation and colonization of the mouse brain with human glial cells, i.e., oligodendrocytes and astrocytes. Intracerebral inoculation of JCPyV isolate Mad-1 led to virus replication of human astrocytes and glial progenitors, along with focal demyelination and gliosis. The oligodendrocytes were only rarely infected, yet exhibited apoptotic death, suggesting that JCPyV kills them by programming them to undergo apoptosis instead of inducing a lytic infection. This is in contrast to PML in humans, where oligodendrocytes are primarily infected and produce progeny virus. Interestingly, the disease pathogenesis was associated with the emergence of virus mutants harboring mutations in the VP1 region, similar to those observed and isolated from PML patients [115,116]. This animal model represents a substantial step forward in modelling JCPyV disease; however, it cannot be used to undertand how JCPyV is transported from the periphery to the central nervous system because JCPyV has been injected into the chimeric mouse brain. Further advances in the development of a humanized model harboring dual engraftments, such as primary human glial progenitor cells and hematopoietic stem cells, may answer this question.

#### 4.3.4. Mouse PyV Infection Model

Similar to human PyV, mouse PyV establish a silent persistent infection in natural populations of mice. Genetic differences exist between mouse and human PyV, for example the mouse PyV genome encodes a third TAg named MTAg, which is the principal oncogene of mouse PyV [117]. At present, none of the human PyV has been shown to express MTAg, except TSPyV, as demonstrated recently by van der Meijden and colleagues [118]. Furthermore, unlike JCPyV and BKPyV, mouse PyV lack the agnoprotein. The observation that PyV-specific CD4+ and CD8+ T cells are induced in mouse PyV infection [119,120] suggested that mouse PyV infection might be a model to study human PyV infection and pathogenesis. In fact, several early studies addressing mouse PyV infection in T cell-deficient mice demonstrated virus-induced demyelination upon productive viral replication in the central nervous system (CNS). However, the use of these mouse models was stopped due to the results of the outcome of mouse PyV infection in nude mice. Nude mice infected intracranially with mouse PyV developed vertebral tumors, which compressed the peripheral nerves and produced paralysis in the absence of demyelination (reviewed in [121]).

Another approach involves mouse PyV infection in mice bearing allogeneic kidneys to mimic BKPyV-associated nephropathy of kidney transplants recipients. In this mouse PyV-renal transplant model, infection with the mouse PyV wildtype strain A2 resulted in a productive replication in the allogeneic kidney graft, severe graft injury and accelerated kidney graft failure [122]. However, results from this mouse model are difficult to extrapolate to kidney transplant recipients since the recipient mice were not immunosuppressed because allogeneic kidneys are not acutely rejected by immunocompetent mice. To address this issue, Albrecht and colleagues performed kidney transplantations in splenectomized and nephrectomized alymphoplasia (aly/aly) mice, which are unable to mount an adaptive immune response [123]. Although persistent high viral loads were observed in aly/aly mice following acute mouse PyV infection, high viral loads were not associated with increased allograft injury or loss of renal grafts suggesting that PyV-associated nephropathy in mice is dependent on an intact adaptive immune response. These findings are in contrast to BKPyV-associated nephropathy, where viral cytopathic changes in tubular or glomerular epithelial cells are central histopathological features. Further studies are necessary to evaluate the utility of mouse PyV-transplant models for the study of BKPyV-associated nephropathy in humans.

#### 4.3.5. Simian PyV SV40 Monkey Model

The simian PyV SV40 infects species of Asian macaque monkeys, especially the rhesus macaque (*Macaca mulatta*). PyV SV40 is closely related to BKPyV and JCPyV, with which it shares approximately 70% sequence homology. As with human PyV, SV40 establishes asymptomatic persistent infections in rhesus macaques. SV40 infection in immunodeficiency virus (SIV)-immunosuppressed rhesus monkey can cause demyelinating brain lesions, analogous to PML in immunocompromised patients, and meningoencephalitis with productive SV40 infection of neurons, which parallels JCPyV neuronal infection in immunosuppressed humans [124,125,126,127]. Thus, rhesus macaques can serve as an important animal model to study JCPyV primary infection and neuropathogenesis.

## 5. Conclusions and Future Perspectives

Available experimental models have so far focused mainly on BKPyV and JCPyV, which allowed important insights into PyV-host cell interaction and recapitulated some pathological features of PyV-associated diseases. However, it seems that we are just looking at the tip of the iceberg. For example, PyV genomes are packaged with histone proteins in infected cells [49,50], suggesting an epigenetic control of PyV gene expression. Knowledge of the epigenetic regulation of PyV genomes may help to understand how PyV persistence is established and maintained. Currently, only MCPyV seems to be associated with cancer, although BKPyV LTAg expression has been reported in early prostate cancer precursor lesions and renourinary carcinomas [128,129]. Potential etiologic roles for other PyV in cancer has to be investigated in appropriate model systems. The increasing use of immunosuppressive or immunomodulatory therapy will undoubtedly increase PyV-associated diseases, and efficient antiviral treatments are severely lacking. To facilitate screening of anti-viral drugs, available cell culture models have to be improved, including infectious recombinant reporter viruses to easily visualize virus-infected cells. Great efforts are needed to develop humanized mice models for other PyV, such as mice engrafted with functional human kidney tissue and immune system, which may allow studies on BKPyV infection and BKPyV-associated nephropathy. Finally, the iceberg under the water seems relatively large when aiming to address the question of the evolution of human PyV and their role as members of the human virome.

## Figures and Tables

**Table 1:** Discovery of human polyomaviruses and associated diseases.

Human Polyomavirus	Abbreviation	Year of Discovery	NCBI RefSeq or GenBank Accession	Source of Isolation	Seroprevalence (%) *	Associated Disease	Ref.
BK polyomavirus	BKPyV	1971	NC_001538	Urine	80–90 ^(a)^	Nephropathy, hemorrhagic cystitis	[2]
JC polyomavirus	JCPyV	1971	NC_001699	Brain	40–55 ^(b)^	Progressive multifocal leukoencephalopathy	[3]
Karolinska Institute polyomavirus	KIPyV	2007	NC_009238	Respiratory tract	55–90	Not known	[4]
Washington University polyomavirus	WUPyV	2007	NC_009539	Respiratory tract	70–90	Not known	[5]
Merkel cell polyomavirus	MCPyV	2008	NC_010277	Skin tumor	60–80	Merkel cell carcinoma	[6]
Human polyomavirus 6	HPyV6	2010	NC_014406	Normal skin	70–75	Not known	[7]
Human polyomavirus 7	HPyV7	2010	NC_014407	Normal skin	35–62	Pruritic rash	[7]
Trichodysplasia spinulosa-associated polyomavirus	TSPyV	2010	NC_014361	Skin lesion	70–84	Trichodysplasia spinulosa	[8]
Human polyomavirus 9	HPyV9	2011	NC_015150	Blood and urine	18–50	Not known	[9]
Malawi polyomavirus	MWPyV	2012	NC_018102	Stool	42–75	Not known	[10]
Human polyomavirus 10	HPyV10	2012	JX262162	Condyloma	99	Not known	[11]
Mexico polyomavirus	MXPyV	2012	JX259273	Stool	Not known	Not known	[12]
St Louis polyomavirus	STLPyV	2012	NC_020106	Stool	70	Not known	[13]
Human polyomavirus 12	HPyV12	2013	NC_020890	Liver	23	Not known	[14]
New Jersey polyomavirus	NJPyV	2013	NC_024118	Muscle biopsy	Not known	Not known	[15]

* References: [14,16,17,18,19,20,21] ^(a)^ Following analysis based on the entire genome or sequence of the major viral capsid protein VP1, BKPyV strains have been classified into four different genotypes (I–IV), corresponding to four serologically-different subtypes. Genotype I is the most prevalent worldwide, while Genotype IV is found solely in East Asia and Europe. In contrast, Genotypes II and III are rarely detected in the human population [22]. ^(b)^ Only one serotype has to date been reported for JCPyV, despite the existence of seven genotypes, numbered 1–8, with Type 5 reclassified as a member of Type 3, and numerous subtypes [23]. European populations typically harbor Types 1 and 4, although Type 2 subtypes have also been described [24,25]. African populations are often associated with Types 3 and 6, with the former also found in Middle-Eastern populations [26], while numerous subtypes from Types 2 (2A, 2B, 2D and 2E) and 7–8 (7C, 8A and 8B) are found in Asia and Oceania [27,28]. Coevolution of JCPyV with human populations is thought to have given rise to the different genotypes and could account for their association with specific ethnic groups [29].
